# Evaluation of the Bleeding Intensity of Patients Anticoagulated with Warfarin or Dabigatran Undergoing Dental Procedures

**DOI:** 10.5935/abc.20180137

**Published:** 2018-09

**Authors:** Marcus Vinicius Santos Andrade, Luciana Azevedo Prata Andrade, Alan Freitas Bispo, Luana de Alencar Freitas, Milena Quadros Sampaio Andrade, Gilson Soares Feitosa, Gilson Soares Feitosa-Filho

**Affiliations:** 1 Escola Bahiana de Medicina e Saúde Pública, Salvador, BA - Brazil; 2 Hospital Santa Izabel da Santa Casa de Misericórdia da Bahia, Salvador, BA - Brazil; 3 Centro de Referência em Doenças Cardiovasculares - Dr. Adriano Pondé, Salvador, BA - Brazil

**Keywords:** Hemorrhage/complications, Anticoagulants, Oral Surgical Procedures, Bleeding Time, Warfarin, Dabigatran

## Abstract

**Background:**

Thrombotic disorders remain one of the leading causes of death in the Western world. Dabigatran appeared as an alternative to warfarin for anticoagulation in the treatment of atrial fibrillation (AF). The risk associated with bleeding due to its use has been documented in several randomized clinical trials, but no large study has examined in detail the risk of bleeding during dental extraction and other dental procedures involving bleeding.

**Objective:**

To compare the intensity of bleeding in individuals taking dabigatran or vitamin K antagonist (warfarin) and undergoing dental procedures.

**Methods:**

Prospective, single-center, controlled study with one single observer. Patients diagnosed with nonvalvular AF, on warfarin or dabigatran, cared for at a cardiology referral center, and requiring single or multiple dental extractions, were evaluated up to seven days post-extraction. The following outcomes were assessed: bleeding time between the beginning and the end of suture and complete hemostasis; bleeding before the procedure, after 24 hours, 48 hours, 7 days, during and after suture removal (late); p<0.05 was defined as of statistical relevance.

**Results:**

We evaluated 37 individuals, 25 in the warfarin group and 12 in the dabigatran group. Age, sex, weight, height, blood pressure, color, schooling, family income and comorbidities were similar between the two groups. Regarding bleeding after 24 hours of the procedure, no one in the dabigatran group had bleeding, whereas 32% in the warfarin group had documented bleeding (p = 0.028). The other variables analyzed did not differ between the groups.

**Conclusions:**

This study suggests that, regarding dental extraction, there is no statistically significant difference in the intensity of bleeding of patients taking dabigatran as compared to those taking warfarin. Bleeding 24 hours after the procedure was less frequent among patients on dabigatran.

## Introduction

Thrombotic disorders remain one of the leading causes of death in the Western world. Several treatments with anticoagulants have been used, including unfractionated heparin, low-molecular-weight heparin, fondaparinux, vitamin K antagonists (warfarin), and novel oral anticoagulants (NOACs), such as apixaban, dabigatran and rivaroxaban.^1^ Warfarin, the major anticoagulant, has been used for more than five decades in the United States and worldwide. Over two million people in the United States are estimated to use warfarin, with approximately 300,000 new prescriptions every year.^2^

Despite their proven efficacy, the clinical use of vitamin K antagonists has some drawbacks, such as food and drug interaction, variable anticoagulation response, slow onset of therapeutic effects, need for therapeutic response monitoring by use of prothrombin time (PT) and International Normalized Ratio (INR), and narrow therapeutic range.^3^ Based on the drawbacks of warfarin use and the low efficiency of anticoagulation rates in clinical practice, studies assessing NOACs have been planned and conducted in recent years.

The NOACs have been developed and properly assessed in phase 2 and 3 studies, which have clearly demonstrated their efficacy and safety. Some drugs are factor IIa inhibitors (thrombin inhibitor), such as dabigatran, while others are factor Xa inhibitors, such as apixaban, rivaroxaban and edoxaban. Patients with atrial fibrillation (AF) are at high risk for stroke. Although warfarin and other vitamin K antagonists are highly effective, reducing the risk of stroke in approximately two thirds of the cases, their use has the already described drawbacks. Recently, NOACs have shown to be as effective as warfarin, or even superior, in preventing stroke and systemic embolism.^4^

Dabigatran etexilate, an oral direct thrombin inhibitor, has a serum half-life of 12 to 17 hours and requires no INR monitoring. In the RE-LY trial,^5^ which proved the non-inferiority and efficacy of that NOAC as compared to warfarin, 10% of the study participants needed to undergo dental procedures.^5^ The RE-LY trial subgroups (dabigatran and warfarin) have shown similar periprocedural bleeding rates, with greater benefits for the dabigatran group regarding major bleedings because of the faster reversion of the drug's effect. Therefore, dabigatran has emerged as an alternative to warfarin for anticoagulation in the treatment of AF and venous thromboembolism.^6^ Several guidelines have validated the use of warfarin or any NOAC (class of recommendation I, level of evidence A) for patients with nonvalvular AF and indication for antithrombotic therapy; however, NOACs are not indicated to patients with mechanical prosthetic valve or hemodynamically significant mitral stenosis, because such patients have been excluded from the major studies on NOACs in AF.^7^

Cardiologists are often sought for guidance regarding the suspension of anticoagulants before a dental procedure, because of the concern with bleeding. In addition, dentists should be aware of the NOACs prescribed, as well as of their peculiarities, to ensure that patients receive safe and proper dental treatment.^8^ The risk for hemorrhagic events associated with the use of NOACs has been documented in several randomized clinical trials, but no large study has assessed specifically the risk for bleeding after a dental extraction or other dental procedure involving bleeding. Dental extraction is one of the most common surgical procedures and can cause significant bleeding. With the increasing use of direct thrombin inhibitors in clinical practice, the occurrence of bleeding and hemorrhagic complications in that context requires better assessment.^9^

The present study aimed at assessing the severity of bleeding associated with the use of dabigatran as compared to traditional oral anticoagulation (warfarin) in individuals undergoing dental procedures.

### Methods and Sample Selection

This is a prospective single-center controlled study with one single observer. Patients diagnosed with nonvalvular AF and indication for anticoagulation, cared for at a cardiology referral center, and requiring single or multiple dental extractions were included. All patients provided written informed consent, and the study protocol was approved by the Ethics Committee in Research of the institution. The patients were followed up by a clinical cardiologist in two groups: group 1, warfarin (25); group 2, dabigatran (12).

Patients, independently of sex, aged 18 years or older, with nonvalvular AF and on an oral anticoagulant (warfarin) or NOAC (dabigatran) were selected. Those on oral dabigatran at the dose of 150mg every 12 hours received the drug from the Municipal Health Department. Dabigatran was specifically chosen because of a previously established care partnership between the Municipal Health Department and Boehringer Ingelheim's laboratories for anticoagulation of patients with nonvalvular AF. Individuals with the following characteristics were excluded: contraindication for anticoagulation; refusal to provide written informed consent; use of warfarin with an INR outside the therapeutic range (2.0 - 3.0) on the day of the dental procedure.

Before the procedure, the patients' vital data, such as systemic blood pressure and heart rate, as well as their weight and height were assessed. In addition, the patients were asked about their race (white, mixed or black), educational level and family income. Those of the warfarin group underwent blood collection to measure PT and INR before the procedure (same day) by use of hemostasis screening tests, while those of the dabigatran group took the predicted dose (150 mg every 12 hours). Patients received prophylaxis for infectious endocarditis, when indicated, in accordance with current guidelines. The dental extractions were performed according to the department's protocol for dental treatment of patients with heart diseases on anticoagulants. The local hemostatic measures comprised appropriate sutures, cellulose sponge and tranexamic acid (ground pill). All patients were prescribed dipyrone, 1 g up to every 6 hours for pain after the procedure, or, in case of allergy to dipyrone, paracetamol, 750mg up to every 6 hours.

**Figure 1:**
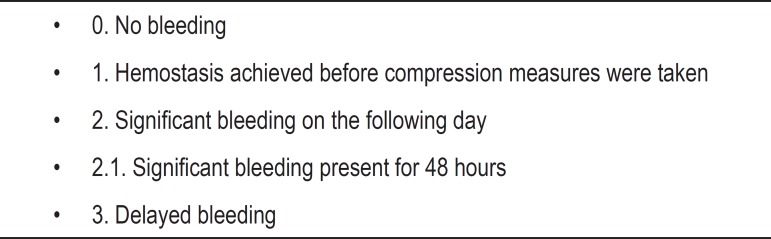
Bleeding scale

Bleedings or hemorrhagic complications of the patients on oral anticoagulants were assessed by the surgical dentist (single observer) during and after the single or multiple dental extractions. Primary outcome was defined as bleeding time 1, between the beginning of suture and complete hemostasis. The following outcomes were also assessed: bleeding time 2 (between the end of suture and complete hemostasis), bleeding before dental extraction, bleeding during dental extraction, and bleeding 24 hours, 48 hours and 7 days after the procedure. The bleeding scale was used, and major bleedings were those from 2.1 on, as described by Iwabuchi et al. (Figure 1).^10^

### Data analysis

#### Statistical analysis

The *Statistical Package for Social Sciences* (SPSS), version 15, was used for data analysis. Nonparametric tests were used for the analysis of continuous variables, because of the small sample size and the well-known low performance of the tests of adherence to normality in small samples. Continuous variables were described as median and interquartile range (IQR). Categorical variables were described as relative frequency and compared by use of Chi-square and Fisher exact tests. Continuous variables were compared by use of Wilcoxon test for dependent samples, while Mann-Whitney U test was used for independent samples. Statistical significance was defined as p value < 0.05.

#### Sample size calculation

The sample size was calculated to yield statistical power of 80% and an alpha of 5%, estimating, based on previous clinical experience, a total bleeding time around 180 ± 60 seconds for the warfarin group, and expecting a reduction of at least 60 seconds in the dabigatran group as compared to the warfarin group. Thus, the sample size calculated was 12 patients in each group. Because the inclusion of patients in the warfarin group was easy, its sample size was doubled.

## Results

### Clinical characteristics of the sample

From January to June 2017, 48 patients with nonvalvular AF were selected, and 11 patients who required no bloody dental procedure were excluded. This study included 37 patients, 19 (51.4%) of the female sex, ages ranging from 34 to 85 years (median, 69 years, IQR: 58-65 years). The patients had multiple comorbidities, such as hypertension (78.4%), diabetes (37.8%), and heart failure (27%). All patients were on regular medical follow-up and on regular use of the drugs prescribed by their attending physicians.

Of the patients included in the study, 25 were selected for the warfarin group and 12, for the dabigatran group (150 mg). When comparing both groups, before the intervention, no significant statistical difference was observed regarding age, sex, race, educational level, family income, systemic blood pressure, heart rate, weight, height and number of teeth to be extracted (Table 1).

**Table 1:** Clinical characteristics of the patients studied according to the intervention group

Variable	Warfarin (n = 25)	Dabigatran (n = 12)	p Value
Age (median, IQR) - years	67 (54.5-75.5)	71 (65.5-80)	0.360
Female sex - n (%)	12 (48)	7 (58.3)	0. 556
SBP (median, IQR) - mm Hg	120 (110-140)	130 (102.5-137.5)	0.810
DBP (median, IQR) - mm Hg	80 (70-85)	80 (62.5-80)	0.432
HR (median, IQR) - bpm	76 (62.5-88)	76.5 (67.5-90.3)	0.554
Weight (median, IQR) - kg	68 (56.5-78.5)	67.5 (60-75.3)	0.810
Height (median, IQR) - m	1.61 (1.49-1.69)	1.605 (1.52-1.70)	0.810
INR (median, IQR)	2.5 (2.2-2.97)	-	-
Teeth extracted (median, IQR)	1 (1-1.5)	1 (1-1.75)	0.962
Black color (%)	10 (40)	06 (50)	0.565
Family income (up to 1 minimum wage) - n (%)	20 (80)	10 (83.3)	0.594
Educational level (incomplete secondary level) - n (%)	16 (64)	7 (58.3)	0.507
Arterial hypertension - n (%)	18 (72)	11 (91.7)	0.177
Diabetes mellitus 2 - n (%)	10 (40)	04 (33.3)	0.493
Heart failure - n (%)	07 (28)	03 (25)	0.588
Traumatic dental extraction - n (%)	05 (20)	03 (33.3)	0.311

IQR: interquartile range; bpm: beats/minute; SBP: systolic blood pressure; DBP: diastolic blood pressure; HR: heart rate; INR: International Normalized Ratio. Continuous variables (expressed as median and 25th and 75th percentiles) were compared by use of Mann-Whitney U test. The categorical variables "sex" and "black skin color" were compared by use of Chi-square test. The other variables were compared by use of Fisher exact test (expected frequencies ≤ 5).

### Clinical outcomes

Regarding the primary outcome, bleeding time 1 showed no statistically significant difference between the groups (median of 300 seconds for both groups). Regarding the other outcomes, such as bleeding time 2, bleeding before dental extraction, bleeding during dental extraction, bleeding 48 hours after the procedure, and the bleeding scale, no significant difference was found. However, bleeding 24 hours after the procedure was not identified in any patient in the dabigatran group, but eight patients in the warfarin group (32%) had it, resulting in a statistically significant difference (p=0.028) between the groups. No significant difference was observed in delayed bleeding, during and after suture removal. Table 2 illustrates the clinical outcomes of bleeding in both groups before and after the intervention.

**Table 2:** Clinical outcomes of bleeding in the warfarin and dabigatran groups before and after dental extraction

Outcome	Warfarin	Dabigatran	p Value
Bleeding time 1 (median, IQR)	300 (240-390)	300 (240-360)	0.597
Bleeding time 2 (median, IQR)	0 (0-60)	0 (0-60)	0.666
Bleeding scale (median, IQR)	1 (1-2)	1 (1-1)	0.124
Bleeding before dental extraction - n (%)	24 (96)	12 (100)	0.676
Bleeding during dental extraction - n (%)	25 (100)	12 (100)	-
Bleeding after 24 hours - n (%)	8 (32)	0	0.028**
Bleeding after 48 hours - n (%)	5 (20)	0	0.122
Delayed bleeding - n (%)	5 (20)	0	0.122
Bleeding during suture removal - n (%)	8 (32)	2 (16.7)	0.285
Bleeding after suture removal - n (%)	0	0	-

(**) p value < 0.05 (-): statistical data not available because either all or no patient had the outcome in the two groups.

Continuous outcomes (expressed as median and 25th and 75th percentiles) were compared by use of Mann-Whitney U test. Categorical outcomes were compared by use of Fisher exact test (frequencies expected ≤ 5).

## Discussion

This study's results show, in individuals submitted to dental extraction, no statistically significant difference in the bleeding intensity of individuals on dabigatran as compared to those on warfarin, but suggest a lower frequency of bleeding 24 hours after the procedure in those on dabigatran.

The InterFib registry has assessed 15,174 patients with AF in 47 countries, including Brazil and South America. When analyzing the data of our region, the rate of an oral anticoagulant use was 45%, of which 44% had INR within the 2-3 therapeutic range. Thus, of the patients with AF and indication for an oral anticoagulant, only 20% were properly anticoagulated.^11^ There has been great controversy regarding the use of anticoagulants in planning dental treatments that involve bleeding. The major concerns about the use of NOACs in invasive dental procedures involving bleeding were the lack of a specific antidote for reversing the medicine effect and the risk of the thrombotic disease for which anticoagulation was indicated.^12^ In April 2017, the Brazilian Sanitary Surveillance Agency (Anvisa) approved the use of idarucizumab in Brazil to reverse anticoagulation in patients on dabigatran. Idarucizumab is a fragment of monoclonal antibody, which, upon injection into the bloodstream, neutralizes dabigatran via direct binding, preventing its anticoagulant effect. It has been widely used in the emergency setting. The results of the RE-VERSE AD study have confirmed the efficacy and safety of that drug. More recent guidelines on the reversion of the effect of NOACs recommend its use.^13-15^ Patients on oral anticoagulants for different reasons, such as AF, need to have their risk for bleeding and complications during a dental procedure assessed. The management of individuals on warfarin who need to undergo invasive dental procedures involving bleeding and/or oral and maxillofacial surgery has been well documented in the literature and follows the recommendations of the III Brazilian Guideline on Perioperative Assessment.^16^ In contrast, there is no clinical trial in the literature providing specific recommendations for patients on NOACs who need to undergo dental procedures.^17^

A recent study on the use of dabigatran and perioperative management has recommended not to suspend that drug in patients submitted to minor procedures, such as dental cleaning, dental extraction, skin biopsy or cataract surgery, and to perform the procedure preferably 10 hours after the ingestion of the last dose to minimize the risk of bleeding.^18^ Another study has recommended not to interrupt NOACs in simple procedures, such as up to three dental extractions, three implantations, radicular scraping and smoothing, and alveoloplasties.^19^ Cohen et al.^20^ have reported that, for more complex periodontal surgery or more than three extractions, the medication should be suspended 48 hours before and reinitiated 24 hours after the procedure in patients with normal renal function. Breik et al.^21^ have suggested that dabigatran or any anticoagulant should only be interrupted before dental procedures after consultation with the patient's attending physician (clinician or cardiologist), who will assess the risk of bleeding *versus* the risk of thrombosis for each patient. For those on dabigatran for AF without a previous stroke, suspending the medicine 24 hours before the procedure is considered relatively safe; however, for patients with a recent history of deep venous thrombosis, pulmonary thromboembolism or embolic stroke, suspending the medicine might be risky.^21^

The clinician should consider that the number of patients taking NOACs is rapidly increasing and that the conflicting findings of several studies have shown that no ideal management has been established for the use of those medicines in patients who need to undergo dental procedures with a high risk for significant bleeding.^22^ More recent data have emphasized that there is no need to suspend dabigatran in dental extraction, and have suggested that, in cases involving the risk for major bleeding, the decision to temporarily interrupt the drug should be individualized and agreed with the attending physician.^23,24^

### Implications

A recent survey has revealed that dentists are well informed about anticoagulation. They, however, tend to overestimate the risk of bleeding, being cautious about their treatment management, which differs in different parts of the world.^25^ A Brazilian systematic review has highlighted the risk of bleeding in individuals taking anticoagulants, as well as the efficacy and safety of dental interventions in that population.^26^ It is worth noting that the present study is pioneer in Brazil on approaching that practice in patients with nonvalvular AF, and its results should be used to foster the understanding of the magnitude of bleeding in that specific population when taking that class of drug.

### Study limitations

The present study has some limitations: impossibility of being double-blind because of the lack of funding to pay for a double-dummy study, with specific placebo for the two drugs tested and their false INR for monitoring in the dabigatran group. In addition, choosing a continuous variable for primary outcome makes the analysis of subtle differences between the groups more objective, allowing a smaller sample size with statistical adequacy; however, that number is small to assess more robust and rare outcomes in this type of intervention.

## Conclusions

This study suggests that, regarding dental extraction, there is no statistically significant difference in the intensity of bleeding of patients taking dabigatran as compared to those taking warfarin. Bleeding 24 hours after the procedure was less frequent among patients on dabigatran.
